# Multi-reflecting time-of-flight mass spectrometry enables high-accuracy metabolite identification in a demonstration study using serum samples from COVID-19 patients

**DOI:** 10.1007/s11306-026-02539-w

**Published:** 2026-10-03

**Authors:** Adam King, Eleanor Matthews, Garth Maker, Robert Trengove, Shaufa Shareef, Stephen Fowler, Timothy Felton, Angela Simpson, Martin Palmer, Lee A. Gethings, Robert S. Plumb, E. N. Clare Mills

**Affiliations:** 1 Waters Corporation, Cheshire, Wilmslow, SK9 4AX UK; 2 Division of Infection, Immunity and Respiratory Medicine, University of Manchester, Manchester, UK; 3 Medical and Molecular Sciences, School of Veterinary and Life Sciences, Murdoch University, South Street, Murdoch, WA 6150 Australia; 4 Curtin Health Innovation Research Institute, Curtin University, Kent St, Bentley, WA 6102 Australia; 5 Wythenshawe Hospital, Manchester University NHS Foundation Trust, Southmoor Road, Manchester, M23 9LT UK; 6 School of Biosciences, University of Surrey, Guildford, UK; 7 Waters Corporation, Milford, MA 01757 USA

**Keywords:** Metabolomics, Lipidomics, Mass spectrometry, Time-of-flight, COVID-19, Biomarkers

## Abstract

**Introduction:**

Accurate metabolite identification remains a major challenge in untargeted metabolomics due to overlapping signals, isomeric compounds, and insufficient mass resolution. The multi-reflecting time-of-flight (MRT) platform provides mass resolution exceeding 200,000 Full Width at Half Maximum and sub-ppm mass accuracy, enabling detailed isotopic fine structure analysis.

**Objectives:**

The performance of the MRT platforms in identifying lipids and metabolites was evaluated using selected serum samples from a group of hospitalized COVID-19 patients.

**Methods:**

Lipids and polar metabolites were extracted from human serum samples from the Manchester Asthma, Allergy and Thoracic Surgery Biobank and analysed using the MRT platform with data-independent acquisition.

**Results:**

The MRT platform facilitated the resolution of overlapping lipid species and improved annotation confidence. In a pilot cohort (*n* = 8), metabolomic differences associated with disease severity were observed, alongside annotation of clinically relevant metabolites including sulphated bile acids and retained iohexol associated with renal dysfunction.

**Conclusions:**

These findings demonstrate that MRT platform enhances confidence in metabolite identification and supports high-throughput metabolomics, offering significant potential for biomarker discovery in clinical studies.

**Supplementary Information:**

The online version contains supplementary material available at https://doi.org/10.1007/s11306-026-02539-w.

## Introduction

Along with transcriptomics and genomics, omics sciences, such as proteomics and metabolomics together with its sub-field, lipidomics, provide information-rich approaches to investigate biological processes. This includes disease biology, drug toxicity, on/off target pharmacology, drug non-responders and changes to the microbiome amongst others (Bauermeister, 2022; Raja, 2021). These ‘omics methods can be applied in different ways, using profiling approaches to map classes of compounds and provide metabolic fingerprint characteristic of a particular state. In contrast in metabonomics the concentrations of metabolites, including molecules that arise from chemical rather than enzymatic processing (also known as metabonates) (Lindon, 2003), are determined over time. The sciences of metabolic profiling, metabonomics, (Beckonert, 2007; Coen, 2003; Fiehn, 2001; Nicholson, 1999) and lipidomics (Fahy, 2005) involve the measurement of changes in endogenous metabolites/lipids resulting from an external influence e.g., disease, genetic condition or toxic insult, etc. Biological samples contain tens of thousands of metabolites/lipids with a broad range of polarity from highly polar amino acids, organic acids and nucleotides to less polar metabolites such as bile acids, steroids and lipids all of which are present over a broad dynamic range (Ding, 2023; Grzybek, 2019).

Untargeted metabolomics/lipidomics usually relies on high resolution mass spectrometry (MS) and either via direct measurement (Gao, 2018) or coupled to a chromatographic technique e.g., liquid chromatography (LC), gas chromatography (GC), supercritical fluid chromatography (SFC) (Shulaev, 2018) or capillary electrophoresis (CE) (Ortiz-Villanueva, 2017) to detect, annotate and identify analytes in the sample. Of these, LC-MS(MS), employing high-resolution MS combined with sub-2 μm particle LC, has become the technique of choice due to its sample compatibility, speed, specificity, analyte resolution and sensitivity (Plumb, 2023).

Although discovery based metabolic profiling has advanced significantly over the last few years (Plumb, 2023), major challenges remain. These include metabolite coverage, feature detection, dynamic range, quantification, peak annotation, and compound identification (Theodoridis, 2023). Modern high-resolution mass spectrometry (HRMS) technologies (e.g., time of flight (ToF) (Mamyrin, 2001), Orbitrap (Zubarev, 2013), Fourier-transform ion cyclotron resonance (FT-ICR) (Marshall, 1998), facilitates the generation of elemental composition via high mass accuracy allowing rapid database searching to create a list of potential markers of interest. However, biological samples contain a number of isobaric or structural isomers which can give rise to several hits for a particular moiety in a database search. Lipids present a particular challenge as a given lipid (e.g., phoshocholine (PC) 34:2) comprises several structurally related isomers with identical m/z values. Additionally, lipids from different classes may possess very similar m/z values. Confidently identifying a unique lipid thus requires high mass resolution to differentiate the lipid species. This task is further complicated by the presence of multiple adduct formation (e.g. H+, Na+, K+) as these adducts can overlap with other coeluting lipids making identification even more challenging.

Unambiguous identification of these metabolites, by LC-MS, requires the generation of “clean” MS1 and MS2 spectra. This can be accomplished by HRMS alone or combination of ion mobility (IM) with HRMS (King, 2020). As previously reported by Jones and co-workers (Jones, 2016), HRMS with resolution above, approximately, 150,000 Full Width at Half Maximum (FWHM) and 1 ppm mass accuracy can significantly simplify database searching, reducing the number of potential hits and facilitating a deeper interrogation of metabolome (Jones, 2016). Many types of high-resolution mass analysers can achieve mass resolution measurements of up to 1,000,000 FWHM or greater but each present their own individual challenges. For example, ion-trap based mass analysers require long acquisition times in order to acquire the highest resolution possible, while resolution of ToF instrumentation is dependent on the distance the ions transition through the vacuum. The extended acquisition times (e.g., 1 s or more) (Lai & Wang, 2023; Rodriguez-Aller, 2013; Urban, 2016) of trap-type instruments present issues when combining them with fast orthogonal separation techniques such as GC, LC, SFC, CE which produce narrow chromatographic peaks (1–3 s), limiting the number of data points that can be collected for each analytical peak or necessitating long chromatographic analysis. ToF based analysers have rates of data acquisition that are compatible with chromatographic separations (Cooper-Shepherd, 2023; Xie, 2012) but increasing the resolution usually means increasing the length of the path that the ions travel, classically achieved by using a longer flight tube. However, increasing the size of the instrumentation is not always logistically practicable due to the requirement for a large amount of laboratory space, and the use of a longer ion path results in a reduction in signal from elongating the ion’s transit.

An alternative approach to increasing the path length is to reflect ions several times within the ion flight tube. Conventional reflectrons contain a grid system, which can interfere with ions transitioning through these grids reducing ion transfer efficiency. These grids have only a marginal effect on the signal quality when performing a few reflections, however when attempting to conduct multiple reflecting events, they can cause considerable reduction in signal intensity as well as impacting mass accuracy. Multi-reflecting time of flight (MRT) represents a new approach to ToF design with these challenges in mind, significantly increasing the length of the ion path with minimal ion loss (Cooper-Shepherd, 2023; Willis, 2021), giving rise to mass resolution values of 200,000 FWHM or more, and acquisition rates of up to 30 Hz. This combination of high mass resolution at speed presents an attractive option for LC-MS based lipidomics and metabolomics.

We have undertaken a demonstration study, using a novel MRT mass spectrometer to profie metabolome/lipidome of human serum samples obtained from a small number of hospital in-patients with COVID-19, selected from the ManARTS (Manchester Allergy, Respiratory, and Thoracic Surgery) Biobank COVID-19 cohort (Shareef et al., 2026).

## Methods

### Materials

All chemicals were obtained at the highest purity available. Deionized water was purchased from Honeywell (Berkshire, UK). Methanol, isopropanol, and acetonitrile were purchased as LCMS grade from Sigma Aldrich (Poole, UK). The mobile phase additives ammonium and formic acid (Sigma Aldrich, Poole, UK) were obtained at analytical reagent grade and LCMS grade, respectively. Methyl-*tert*-butyl ether (MTBE) (Sigma Aldrich, Poole, UK) was obtained as HPLC reagent grade. Mass Spectrometry calibration solution consisting of sodium formate and the lock mass solution of Leucine enkephalin was obtained from Waters Corporation (Wilmslow, UK).

### Patient samples

Samples of human serum were obtained from 8 patients who were recruited into the ManARTS Biobank following diagnosis with COVID-19 as an inpatient at a Greater Manchester hospital (Shareef et al., 2026), demographic characteristics of whom are summarized in Table 1. Study approval was provided by ManARTS Biobank, where patients provided their informed consent in accordance with the Human Tissue Authority and the National Research Ethics Service (ethical approval REC ref: M2020-92). ManARTS Biobank is funded by the National Institute for Health Research (NREC 15/NW/0409).

**Table 1:** Summary of the demographic characteristics of ManARTS COVID-19 patients selected for the study

Characteristic		*N* (%)	Characteristic		*N* (%)
Sex	Female	1 (12.5)	Infection wave	Wave 1 (10-MAR-2020 to 02-SEP-2020)	5 (62.5)
	Male	7 (87.5)		Wave 2 (03-SEP-2020 to 10-JAN-2021)	3 (37.5)
Age	Mean (Median)	67 (66.5)	BMI	≤ 25 (Normal)	2 (25)
				Overweight (25–29.9)	4 (50)
Comorbidities	Hypertension	4 (50)		Obese (BMI ≥ 30)	2 (25)
	Hypercholesterolemia	1 (12.5)			
	Diabetes	4 (50)			

The patients were subsequently characterized into two phenotype groups based on disease severity, “mild” and “severe.” Patients were assigned to phenotype groups based on their Manchester COVID-19 Severity Score (MCSS), an in-house severity score and clinical management tool, which was determined by clinicians at the time of treatment (Mann, 2020; Shuwa, 2021). MCSS was determined each day of the patient’s hospital stay post-recruitment, and the maximum MCSS was used. MCSS is defined by the level of intervention required, with factors including admission to the intensive care unit (ICU), need for/type of ventilation, and where no ventilation was required, FiO_2_ required to maintain target oxygen saturation. Where sufficient data were available, the WHO COVID-19 severity score was applied (retrospectively) (World Health Organization, 2022). The serum samples were stored at −80 °C prior to sample preparation. The severity of infection and infection status (active/convalescent) is summarized in Table 2.

**Table 2:** Summary of the COVID-19 infection status of the subset of patients whose samples were used in this study

Patient No.	Days from COVID-19 diagnosis to sampling	Maximum Manchester COVID-19 severity score	WHO severity score	COVID-19 infection status at time of sampling
Group 1: mild active infection/convalescent				
6	5	Mild	Not assigned	Active
2	16	Mild	Not assigned	Convalescent
3	139	Mild	Not assigned	Convalescent
Group 2: severe, active infection				
4	1	Severe	Not assigned	Active
5	2	Severe	Severe	Active
7	2	Severe	Critical	Active
8	7	Severe	Critical	Active
1	18	Severe	Not assigned	Active

### Sample preparation for mass spectrometry

Samples were thawed at 4–8 °C. A study quality control sample (QC) was prepared by combining 10 µL of each study sample prior to extraction. The QC sample was prepared for analysis together with individual patient serum samples, as previously described (Matyash, 2008). Serum (30 µL, in duplicate) and a process blank (water) were mixed with 63 µL of ice-cold methanol, followed by 250 µL of MTBE. The resulting solutions were vortex mixed and incubated at 4–8 °C for 2 h to ensure full protein precipitation. 138 µL of water was added to each sample to facilitate phase separation. Samples were then vortex mixed for 15 s and centrifuged for at 4,500 x g for 10 min at 4 °C to pellet precipitated proteins and separate aqueous and organic phases. The aqueous phase was removed and transferred to separate nitrogen-flushed vials, then dried under nitrogen before being reconstituted in 100 µL acetonitrile : water (90:10, v: v) prior to analysis. The upper organic lipid phase was discarded.

### Mass spectrometry analysis

LC-MS analysis was undertaken using an ACQUITY™ UPLC™ I-class chromatography system (Waters Corporation, Milford, MA, USA) attached to a SELECT SERIES™ MRT mass spectrometer (Waters Corporation, Wilmslow, Cheshire, UK) and equipped with an ACQUITY BEH™ amide column (2.1 × 100 mm, 1.7 μm) (Waters Corporation, Milford, MA, USA). The column was maintained at 40 °C. Eluant A comprised of water: acetonitrile (95:5 v/v), whilst eluant B comprised of water: acetonitrile (5:95 v/v), both A and B contained 0.1% formic acid and 10 mM ammonium formate. Compounds were eluted using a multi-step linear gradient over 10 min at a flow rate of 0.7 mL/min, with a mobile phase starting composition of 100% eluant B, which was held for 0.1 min before decreasing to 80% B at 5 min, and then 50% B by 6 min, held for 0.5 min before returning to 100% B at 7 min. The column was then re-equilibrated for 3 min at 100% B prior to the injection of the next sample. The extracts were kept at 8 °C and 5 µL of each sample was injected onto the column. The MRT was operated in continuum mode using the data independent acquisition (DIA), MS^E^ (Bateman, 2002; Plumb, 2006), in both positive and negative electrospray ionisation (ESI) polarities at 10 Hz. In this mode of acquisition, the data are collected in alternating functions of low collision energy (precursor) and an elevated collision energy ramp from 20 to 40 eV for fragmentation data. Data were acquired over the mass range of 50–2400 m/z with data acquisition rate of 10 Hz. The ToF analyser was calibrated over the designated mass range using sodium formate (0.5 mM). The MRT mass spectrometer was operated in encoded frequent pushing (EFP) mode (Cooper-Shepherd, 2023; Willis, 2021) to provide the highest mass resolution and reduce multiplexing of the data. The ESI capillary voltage was set to 2.0 kV for both polarities, and the source and desolvation temperatures were set to 120 °C and 500 °C, respectively. Nitrogen flow rates of 50 and 800 L/hr were employed for cone and desolvation gases, respectively. The synthetic peptide leucine enkephalin was employed as the lock spray solution at a concentration of 200 pg/µL and introduced at a flow rate of 10 µL/min through the reference sprayer. The lock spray channel was sampled every 30 s throughout the course of the acquisition. Samples from 8 individuals (extracted in duplicate, and each extract analysed in triplicate) and a pooled QC, were analysed as described in Supplementary Table S3. Six replicate injections of the study QC sample followed by a blank sample extraction were made at the start of the analysis of study analytical batch, and the study QC sample was analysed once in every 10 injections.

### Mass spectrometry data processing

All raw data were acquired using MassLynx™ version 4.2 (Waters Corporation, Wilmslow, UK). Using Progenesis™ QI (Waters Corporation, Wilmslow, UK), the data were then subject to pre-processing, comprising retention time alignment against a QC injection from the middle of the experiment, peak picking, and normalization to all compounds prior to statistical analysis. Data were exported from Progenesis QI and cleaned in R 4.2.1 (Team, 2024), using tidyr and dplyr (Wickham, 2023, 2024). Features appearing in only 1 of 2 biological replicates, or less than 2 of 3 technical replicates were removed. Feature filtering was performed using the pooled QC sample, whereby features with a minimum % CV of > 30 were removed. Compound annotations were carried out using database searching of LIPID MAPS (Conroy, 2024; Fahy, 2009), Metlin MS/MS (Scripps Research Institute, CA, USA) (Guijas, 2018; Montenegro-Burke, 2020) and HMDB (Wishart, 2018, 2022, 2007).The data cleaning pipeline deployed is summarised in Supplementary Table S4 and its impact on the distribution of coefficients of variance in Figure S4.

### Statistical analyses

Statistical comparisons were made between patient groups using the Wilcoxon rank-sum test, with a Benjamini-Hochberg correction for multiple testing (Supplementary Information Section S2). Processed data were further analysed using MetaboAnalyst 6.0, after Pareto scaling (Pang, 2024). Metabolites were clustered hierarchically by Ward’s method prior to presenting as a heatmap. Data were plotted in R 4.2.1 using ggplot2 (Team, 2024; Wickham, 2016). Significantly altered compounds identified by Wilcoxon rank-sum and principal components analysis (PCA) loadings from lipidomics data were annotated using LIPID MAPS, Human Metabolome Database (HMBD), and Progenesis MetaScope. Further details of data analysis are provided in Supplementary Methods Section S1.

### Spectral simulations

High resolution mass spectrum simulations were produced by the publicly available software enviPat isotope pattern calculator (Loos, 2015) (Eawag, Aquatic Research, Dübendorf, Switzerland).

## Results and discussion

### Improving mass resolution using multireflection time-of-flight mass spectrometry

In order to overcome the limitation in mass resolution provided by conventional (flight tube) ToF mass spectrometers, the MRT employs components known as reflectrons (ion mirrors) that utilize voltages to reflect the ion beam back along the length of the tube, doubling the distance the ions travelled and hence increasing the separation (Supplementary Figure S2) (Mamyrin, 2001). Further advances in this technology allowed for multiple reflections of the ion beam to further extend the distance ions travelled and consequently, improved mass resolution (Kaewnarin, 2021; Willis, 2021) whilst minimizing loss of signal intensity.

The MRT mass spectrometer has been designed to operate with either an electrospray, desorption electrospray ionization or matrix assisted laser desorption ionization inlet, after which the ions pass into the vacuum region of the MS where neutral molecules are separated from the ions using a “step wave” ion guide (Giles, 2019). The resulting ions are then transferred to a quadrupole region (Q 1) which can be operated in a wideband pass-through mode or used to select specific m/z values. The ions are then transferred to a collision cell where they can be fragmented or transition through unchanged. Following the collision cell, transfer and focus optics direct the ions for injection into the multi-reflecting time of flight region of the mass spectrometer, which contains a series of gridless reflectron mirrors (Supplementary Figure S2). These gridless mirrors enable the ion flight path to be increased to 47 m without the need to significantly increase the instrument size. Upon each reflection, the ion beam is re-focused through a series of periodic lenses maintaining signal transmission throughout the ToF analysis (Cooper-Shepherd, 2023). Following separation in the ToF regions the ions are directed to a microchannel plate (MCP) detector to record arrival time and signal intensity.

### Pilot analysis of biomarkers of COVID-19 infection

A pilot study was undertaken to assess the ability of this MS platform technology to support the identification of disease biomarkers using a subset of serum samples from eight patients drawn from the ManARTS COVID-19 cohort (Shareef et al., 2026). Their demographic characteristics were representative of the cohort as a whole, being dominated by those over the age of 65, of male gender and a significant proportion who were overweight or obese and had significant comorbidities (Table 1). Patients showed a range of infection severity (Table 2), with a variety of clinical journeys convalescent patients and those who were admitted to ICU (Supplementary Figure S1). These samples were used to explore the application of the MRT platform for serum metabolomics analysis.

### Compatibility with ultra-high performance liquid chromatography separations

**Fig. 1:**
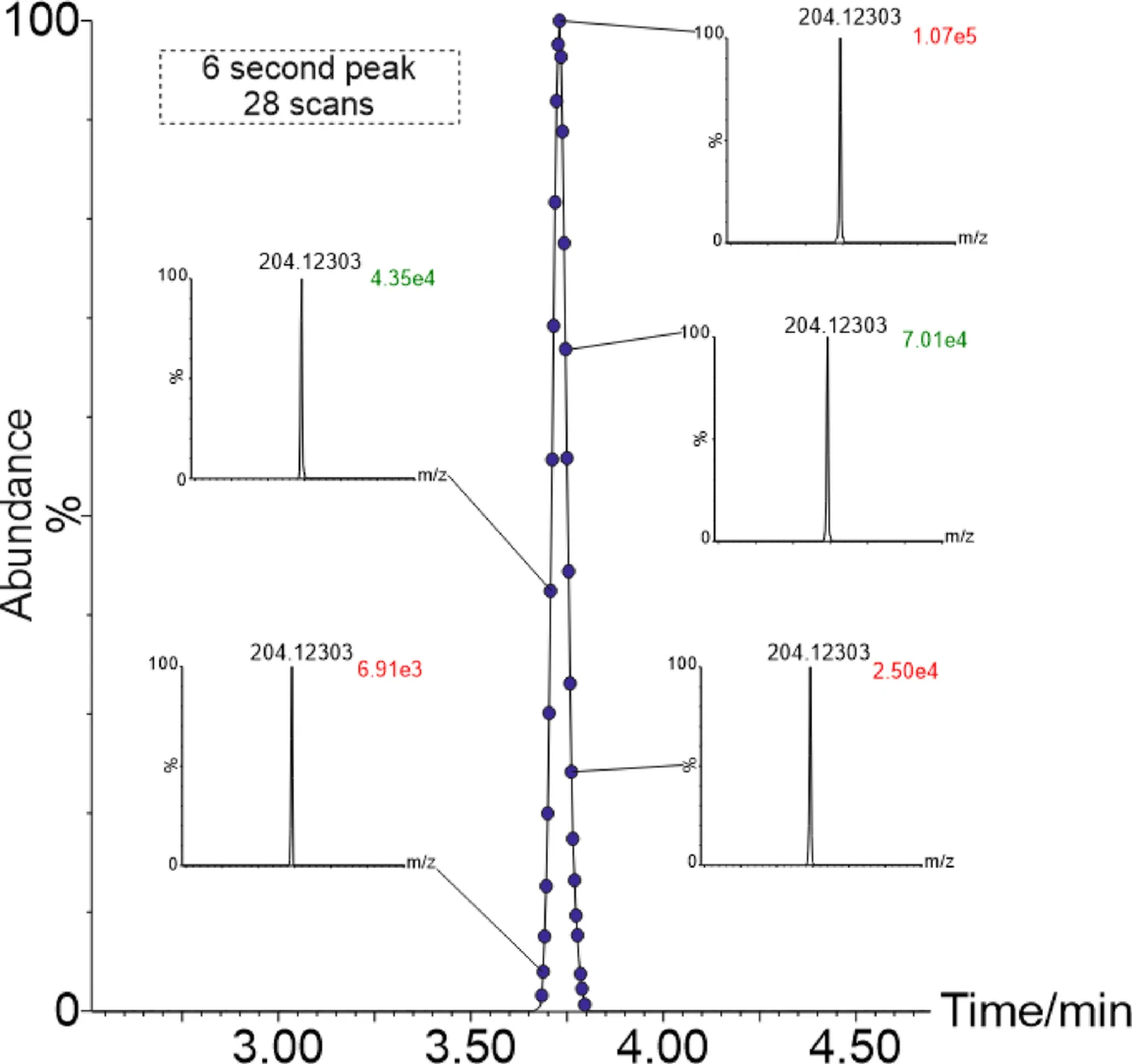
Extracted ion chromatogram of an endogenous acetyl-L-carnitine showing the number of data points collected across the peak. Inserted is an example of an individual scan spectra from varying intensities, which highlight the stable mass accuracy

One of the challenges in undertaking biomarker discovery using metabolomics is the time of analysis, which is largely driven by the requirements of the MS platform employed. Ultra-high performance liquid chromatography (U(H)PLC) has several advantages in that it provides highly efficient and fast separations (Castro-Perez, 2005; Wilson, 2005), providing an attractive balance between throughput, data quality and feature detection in large batch metabolomic studies (Lewis, 2016; Want, 2010). These U(H)PLC analyses produce chromatographic peaks of only a few seconds wide at the base (Munjoma, 2022), therefore, for effective LC-MS/MS metabolomics analysis, it is important for the MS detector to maintain mass resolution and accuracy whilst operating with sufficient speed to provide adequate definition of the analyte peaks. This means that for a U(H)PLC peak with a typical width of Pwb = 2–3 s at the base an acquisition rate of 5–10 Hz is required to provide 8–10 points across the LC peak. Mass spectrometers such as fourier transform MS (FTMS) and ion traps require longer scan times (~ 1 s) to achieve the mass resolution and accuracy required for efficient annotation and identification of mass features. As a consequence, such platforms also require longer LC analysis methods to obtain a sufficient number of points to accurately define the peak height (Urban, 2016). In contrast the design of ToF mass analysers allows for rapid acquisition rates without loss in mass resolution. Indeed, the MRT MS can operate with acquisition rates of up to 20 spectra/sec. The extracted ion chromatogram of the feature annotated as acetyl-L-carnitine (retention time = 3.73 min, m/z = 204.12303) was acquired using the MRT mass spectrometer operated with an acquisition rate of 10 Hz (Fig. 1). The derived data showed that a total of 28 data points were collected across the 6 s wide HILIC peak (even with narrower reversed – phase LC peaks (typically Pwb = 2–3 s) there would be 10–14 points defining the LC peak), thus, providing excellent peak definition and allowing for accurate determination of peak area/response. The precise definition of the LC peaks enables the detection of closely eluting features, demonstrating the potential for the instrument to be utilized for not only qualitative analysis, but also quantitative analysis based on analytical guidance (Center for Drug Evaluation and Research & Research, 2024) for minimum peak data points for quantification.

The reproducibility of mass measurement at these rapid acquisition rates was investigated by the analysis of several data points (*n* = 5) across the LC peak for the mass feature identified as acetyl-L-carnitine (specifically peak lift off, rising edge, apex, and trailing edge of peak) (Table 3; Fig. 1). The derived mass measurements show identical values irrespective of the sampling occasion or signal intensities (6.9e3 to 1.0e5), thus illustrating the stability of the MS mass measurement. As previously mentioned, in comparison with other FTMS type instruments the resolution needs to be reduced to 22,000 FWHM to obtain an acquisition speed of 22 Hz (Scientific, 2020), thus scientists would have to choose between operating at 240 K with only 3–4 points across a 6- sec wide peak, or improved peak definition with reduced mass resolution.

### Resolving co-eluting features and fine isotope distributions

Multiple researchers have reported that the mammalian plasma/serum metabolome contains several thousand of metabolites reflected in the latest version of the HMDB (Wishart, 2022), the sheer number of features means that the coelution of metabolites is inevitable when using conventional LC methods (10–60 min in duration). Consequently any chromatographic peak may give rise to the MS spectra from multiple analytse, which need to be deconvolved for confident peak identification. The presence of heavy isotopes (e.g., 13 C, 15 N, 34 S) further complicates the task of unequivocal peak identification as these isotope peaks from one species can overlap and interfere with the MS spectra of a second analyte resulting in erroneous structural identifications. However, the presence of these heavy isotopes in metabolites can assist in confirmation of molecular formula, based on the fine isotopic patterns (Ding, 2023; Miladinovic, 2012; Nagao, 2014), providing an extra level of confidence for compound annotation. The unique distribution of isotope abundance of each element generates a diagnostic fingerprint, for example nitrogen forms two major isotopes 14 N and 15 N which have relative abundance of 99.6 and 0.4, whereas oxygen has three major stable isotopes 16O, 17O and 18O with relative abundances of 99.757, 0.038 and 0.205, carbon has two major stable isotopes 12 C and 13 C with relative abundances of 98.93 and 1.07 and sulphur has three isotopes 32 S, 33 S, 34 S with relative abundances of 94.93, 0.76 and 4.29. When these atoms are combined to form metabolites or lipids, they create a unique “fine isotope” pattern in the MS spectrum based on the constituent atoms and their proportions, and this provides an extra level of information which can be exploited to simplify the identification of unknown metabolites/lipids with very similar accurate masses. Detecting and taking advantage of this “fine isotope” detail requires sufficient resolution in the mass spectrometer signal to separate and identify these isotopes and record them, with a high degree of accuracy.

**Fig. 2:**
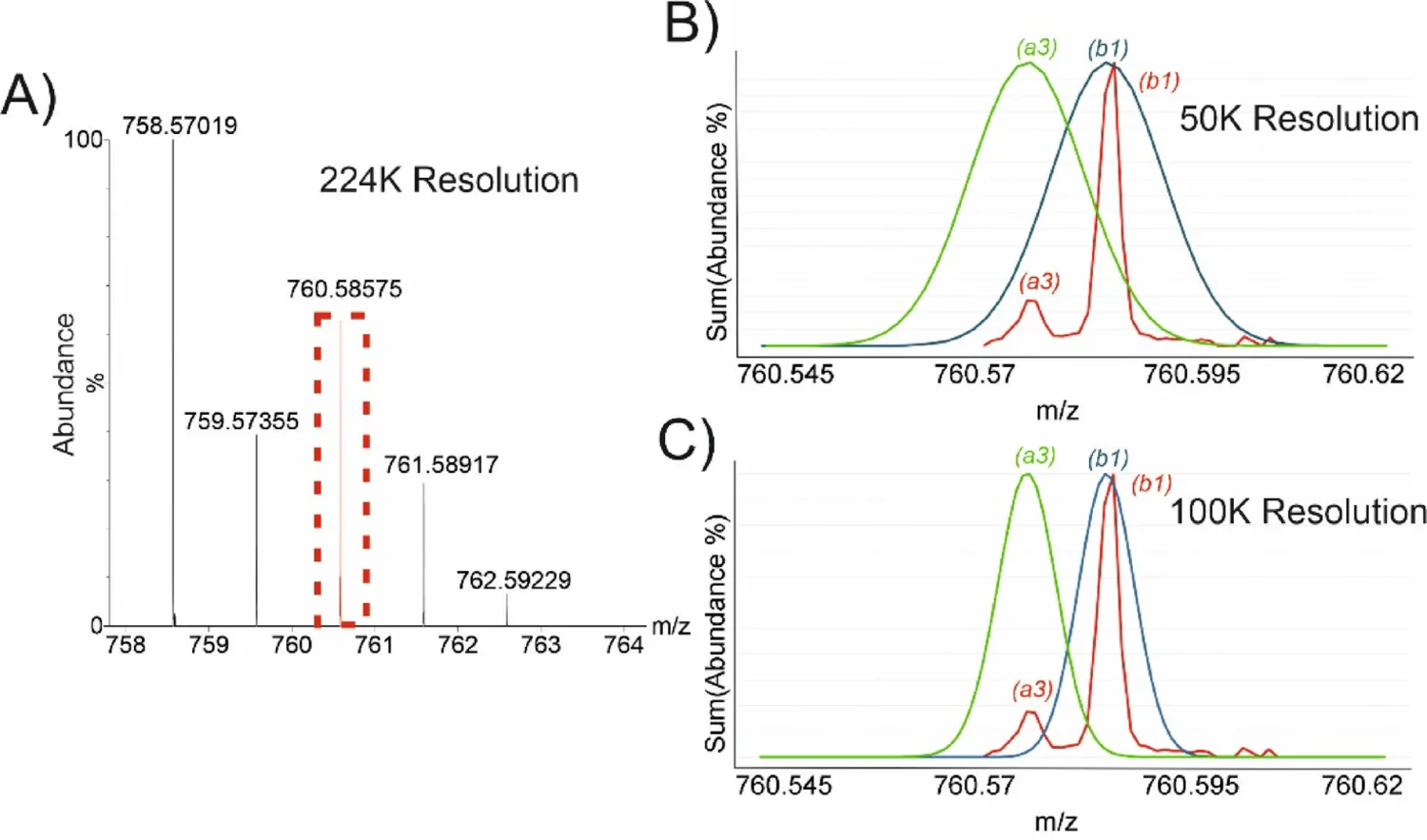
Mass spectra of two co-eluting lipids. Mass features eluting at 1.5 min with highlighted isotopic region in red **(A)**, overlaid raw fine isotopic spectral data (red) with simulated ToF MS operating at 50,000 FWHM **(B)** and 100,000 FWHM (**C**) for the two lipids, PC 34:2 (green) and PC 34:1 (blue) showing the ability to resolve the a3 isotope peak from the b1 monoisotopic peaks for PC 34:2 and PC 34:1 respectively

The large number of lipid species present in a plasma, serum or tissue sample means that there is a high probability of the coelution of lipids with either very similar m/z values or similar m/z values for the heavy isotope (13 C) of one lipid and standard isotope of another (12 C). Due to the biphasic extraction protocol used, a proportion of the aqueous phase contains some lipid species (Vale et al., 2019), including some of the PCs, which were used to demonstrate the platform characteristics. The high mass measurement characteristics, resolution, and mass accuracy, of the MRT instrumentation should allow for the isotopic pattern of an analyte be accurately determined and fine isotope detail recorded, thus allowing co-eluting analytes with similar accurate masses to be differentiated based on their fine isotope patterns. To evaluate the potential of this new MRT platform for lipid isotope deconvolution, the MS/MS data for the analysis of several co-eluting lipids was investigated. The data shown in Fig. 2 illustrates the low collision energy spectra (MS1) obtained from the chromatographic peak eluting with a retention time of 1.5 min) analysis of the human serum extract acquired on the MRT instrument platform in positive ESI mode.

The combined spectrum for the LC peak contained 4 major peaks, m/z = 758.57019, 759.57355, 760.58757 and 761.58917. The molecular ion m/z = 758.57019 (Fig. 4A) was annotated as PC (34:2) using the LIPID MAPS database searching (employing a threshold of +/- 0.01 m/z). A close inspection of the next most intense peak in the spectra (m/z = 760.58575) revealed the presence of two peaks ((1) m/z = 760.57678 and (2) m/z = 760.58575), the mass resolution of the peak was determined to be 224,770 FWHM. These results suggest that the smaller first peak (m/z = 760.57678) is the 13C2 isotope of PC (34:2) and that the second peak (m/z = 760.58575) is likely due to the presence of an additional co-eluting lipid which, following database searching, returned a possible annotation of PC (34:1) (Table 3. For comparison, the simulated MS spectrum obtained from a conventional QTof type instrument operating at 50,000 and 100,000 FWHM resolution is given in Figs. 2B and C, respectively. This data shows that even at 100,000 FWHM resolution it was not possible to detect the presence of two separate isotopes as they were unresolved).

**Fig. 3:**
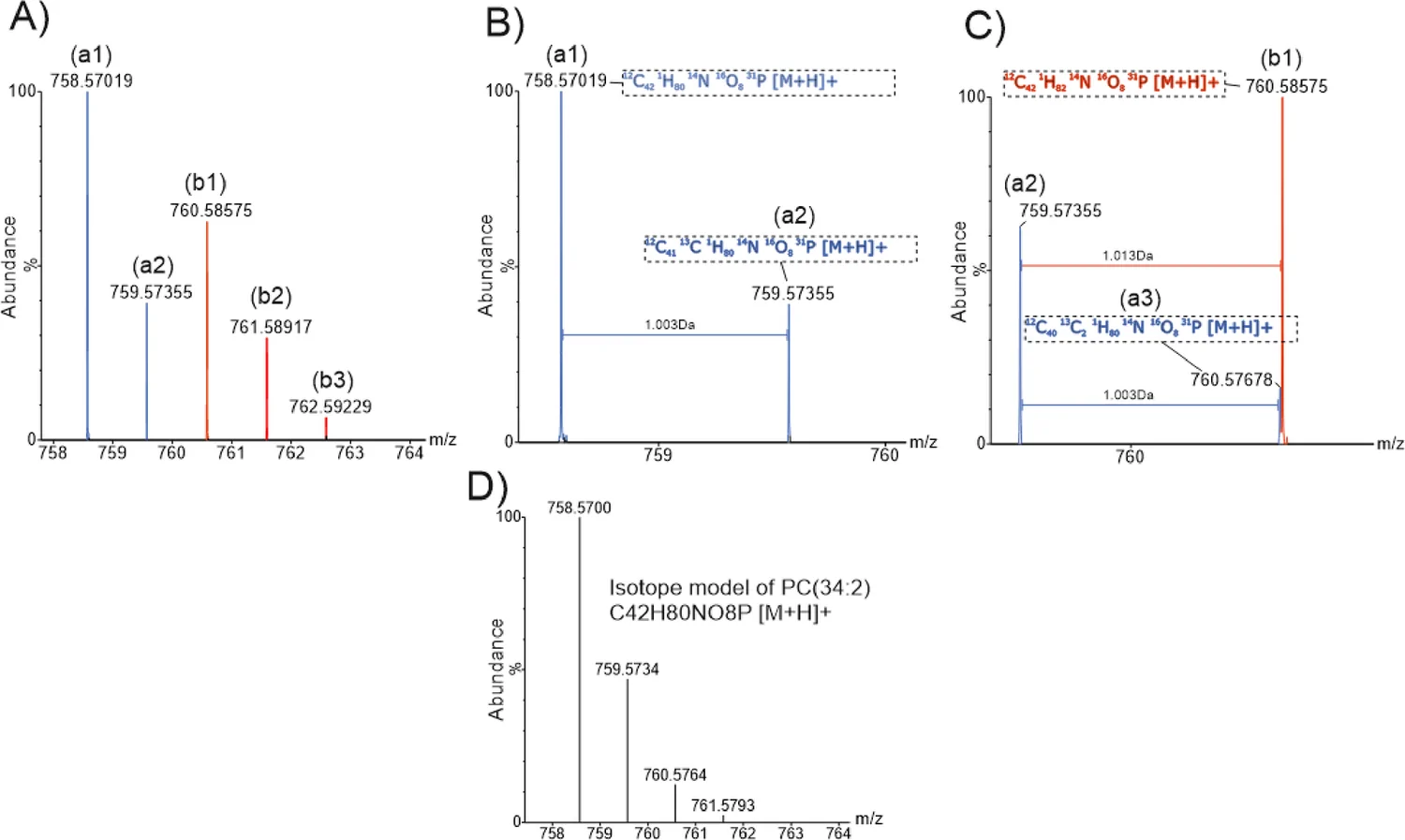
Precursor ion and isotopic spectra of selected lipid features. **A** The isotopic region for mass features eluting at 1.5 min; m/z = 758.57019 – annotated as PC 34:2 and its 13C2 isotope of PC 34:2, m/z = 760.57678 (blue) and PC 34:1, m/z = 760.58575 (red); **B** Highlighted spectra for PC 34:2 and its 13C2 isotope; **C** Highlighted spectra for putative PC 34:1 (m/z = 760.58575) (red) and a second 13C2 isotope of PC 34:2, m/z = 760.57678 (blue); **D** Model isotope distribution for PC(34:2)

Further inspection of the mass difference between each ion in the MS spectrum for these two compounds shows there are several distinct/unique MS1 peaks observed in the mass region m/z 758–763 (Fig. 3A). The mass difference between the two peaks labelled a1 and a2 (Fig. 5A) was 1.003 Da. The peak a1 was annotated as the base peak for PC (34:2) with a corresponding elemental composition of 12C42 1H80 14 N 16O8 31P and a2 corresponds to the presence of a 13 C isotope of PC (34:2) with the elemental composition of 12C41 13 C 1H80 14 N 16O8 31P. In addition, there is also a small third isotopic peak also visible (a3) with a mass measurement of 760.57678, the mass difference between ions a2 and a3 was determined to be 1.003 Da, which would correspond to the presence of a second 13 C isotope (12C40 13C2 1H80 14 N 16O8 31P) (Fig. 3C). In contrast, the mass difference between ions a2 and b1 was determined to be 1.013 Da, which is 0.01Da greater than the presence of an additional 13 C isotope, suggesting that this ion belongs to a separate lipid feature, unrelated to PC (34:2). The theoretical isotope model for PC (34:2), calculated at a FWHM resolution of 200,000, is given in Fig. 3D shows the theoretical model and experimental data were consistent with one another. As can be seen from Fig. 3D the HRMS data acquired and the show good agreement.

The small mass differences between the a3 and b1 ion shown in Fig. 3 further illustrates the challenges faced in accurate annotation and confident identification of lipids. Positive ion ESI analysis of the lipid PC (34:2) eluting at a retention time of 1.5 min (Supplementary Figure S5) gave rise to a MS1 peak with an accurate mass value of m/z = 796.52606, corresponding to the potassium adduct of the lipid with the elemental composition 12C421H8014N16O831P [M+39 K]+ (Supplementary Figure S5A). Reviewing the isotopic pattern of this peak, between m/z = 796 and 801, revealed the presence of five major peaks. Careful analysis of the second most intense peak in the spectra (m/z = 798.54163) revealed three distinct signals, m/z = 798.54163, m/z = 798.53253 and m/z = 798.52423 (Supplementary Figure S5B). These can be rationalized as the elemental compositions 12C421H8214N16O831P [M+39 K]+, 12C4013C21H8014N16O831P [M+39 K] + and 12C421H8014N16O831P [M+41 K]+ respectively. These data show that the isotope peak with m/z = 798.54163 is derived from two lipids C42H82NO8P [M + K] + and that of C42H80NO8P [M + K]+. These two lipids were rationalized as PC (34:1) (m/z = 798.54163) and PC (34:2) (m/z = 798.53253 and m/z = 798.52423) formed from combinations of heavy and light potassium and carbon isotopes. The simulated continuum spectrum, generated at a resolution of 200,000 FWHM, for the PC (34:1) and PC (34:2) is again consistent with the experimental data (Supplementary Figure S5C).

### Comparison of metabolomic profiles in patients with COVID-19

**Fig. 4:**
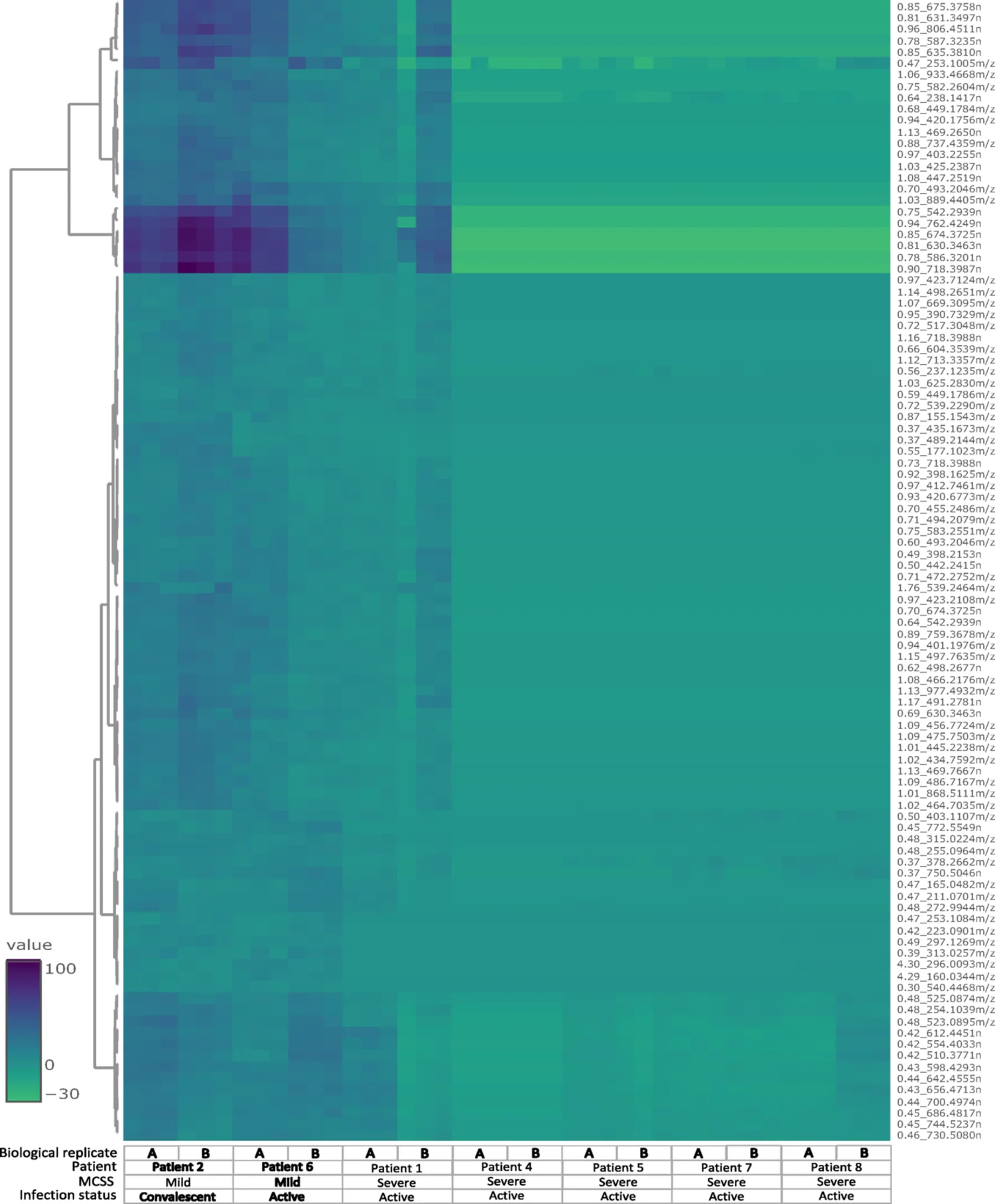
Heatmap of the top 100 features discriminating between r patients with either a severe, active COVID-19 infection or who were either convalescent from mild COVID-19 or had a mild active COVID-19 infection. Colour intensity indicates the Z-score of each feature (row). Each column represents the metabolite intensities within a technical replicate (injection) of a sample. Metabolites were clustered hierarchically by Ward’s method. Samples are displayed without clustering, revealing the natural contrast between patients. Generated in MetaboAnalyst 6.0

The curated MS data for all the patient samples were initially reviewed through PCA to evaluate the technical reproducibility of the measurements which showed poor replication of samples analysed from patient 3 which were therefore excluded from further analysis. The remaining samples showed individual injections clustered closely together at the centre of the sample set, with no evidence of study related drift during analysis (Supplementary Figure S3). Following Kruskal–Wallis test the top 100 mass spectral features discriminating between the patients with either a current severe COVID-19 infection or who were convalescent/had mild current infections identified in positive ionisation mode were then subject to hierarchical clustering by Ward’s method and visualised as a heatmap (Fig. 4). This demonstrates that within this small, proof-of-concept study, the serumlipidome of a convalescent patient who had recovered from a mild infection (patient 2) clustered with that of a patient with a mild, current infection (patient 6). All but one of the patients with severe, active infection (patients 4, 5, 7, and 8) show a broadly similar with (in general) a lower abundance of mass spectral features compared to the serum lipidome of patients 2 and 6. However, the serum lipidome for patient 1, who had a severe, active infection, clustered more closely to the mild/convalescent patients than the other severely ill patients. Within this sub-cohort, patient 1 is the only non-surviving member.

### Compound annotation in human serum facilitated by high mass accuracy

The majority of mass spectral features which have potential to act as disease biomarkers are not annotated (Nguyen et al., 2024). This limits the value of metabolomics in biomarker discovery, since the most robust and reliable disease biomarkers are those which play a role in disease pathogenesis and are not simply correlative in nature. The superior mass accuracy of the MRT was exploited in order to improve mass feature annotation and identification using compound database searching compared to higher mass measurement errors obtained from conventional accurate mass instruments. These conventional HRMS instruments necessitate the use of wider mass tolerances in database queries for feature identification (to minimize the potential of missing features), with the commensurate increase in the number of potential identifications that require curating. This is illustrated by the mass features identified in positive ionization mode and corresponding database annotations are given (Table 3), measured m/z value and calculated theoretical m/z values were used to determine the mass measurement errors. Previous experiments based on different small molecule sample sets have demonstrated the mass accuracy stability provided by the instrument over time (Supplementary Table S1 and S2). Analysis of the data showed that the mass measurement errors ranged between 21 and 134 parts per billion (ppb) (0.021–0.134 ppm), for taurochenodeoxycholate-7-sulfate and phosphatidylcholine (38:3) respectively. These data can be compared to that acquired on a conventional Quadrupole time of flight (QToF), where mass measurement errors typically range between 1 and 3 ppm (Géhin & Holman, 2021; Lai & Wang, 2023) and quadrupole orbitrap type instruments where mass measurement errors of 1–2 ppm have been reported when operating at 240,000 resolution (FWHM) with cycle time of 1.8 s (Kiyonami et al., 2016).

**Fig. 5:**
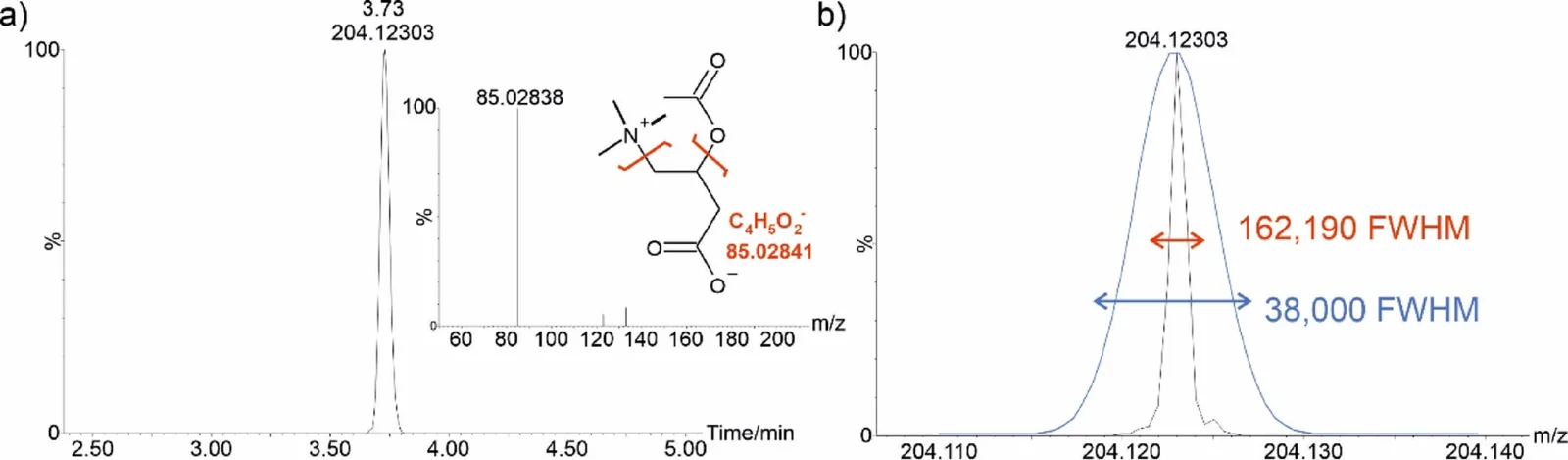
Identification of acetyl-L-carnitine facilitated by high mass accuracy. **a** Extracted ion chromatogram of acetyl-L-carnitine in human serum with elevated collision energy spectra; **b** Comparison of spectral raw data for the acetyl-L-carnitine precursor ion at m/z 204.12303 overlaid with modelled profile data of ion m/z = 204.12303 at a resolutIon of 38,000 FWHM

The mass resolution of the MRT platform allowed identification of the mass feature eluting with a retention time of 3.73 min and an m/z = 204.12303, producing a FWHM resolution of 162,190 (Fig. 5a, b). The resulting high collision energy analysis (MS2) gave rise to the major product ion m/z¬ = 85.02838 (Fig. 5a). Based on the accurate mass and elevated collision energy spectra the metabolite was annotated as acetyl-L-carnitine. For comparison, the simulated MS spectra for a conventional ToF MS operating in sensitivity mode operating at a resolution of 38,000 FWHM is given as the blue spectra overlaid with the measured spectra (Fig. 5b). The data illustrates the reduction in MS peak width collected by the MRT which returned a mass accuracy of 0.03 ppm for MS1 and 0.35 ppm for MS2. This represents a significant improvement over conventional ToF instrumentation which typically report a mass accuracy of 1–3 ppm (Rockwood et al., 2018). The improved mass accuracy also allowed identification of several other mass features using database searching using narrower mass error filters. Thus, the m/z + 597.28739 returned 9 potential identifications when searching using a 2 ppm mass error which could be reduced to only two when the mass error filter was reduced to 0.5 ppm which corresponded to isomeric forms of taurochenodeoxycholate sulphate, which MS and MS/MS alone cannot differentiate.

**Table 3:** Mass accuracy of exemplar compounds annotated in COVID-19 patient serum samples in positive ionization mode

Annotation	Elemental formula	Adduct	Neutral mass (Da)	m/z	Mass error (ppm)	Retention time (min)	MSI level*
PC (34:2)	C42H80NO8P	M + H	757.562155053	758.57019	0.100526	1.5	2
PC (34:1)	C42H82NO8P	M + H	759.577805117	760.58575	0.079911	1.5	2
Taurochenodeoxycholate-sulphate	C26H45NO9S2	M+NH4 M + K	579.2535461	597.28737	−0.048122	3.24	2
Glycoursodeoxycholic acid	C26H43NO5	M + H, M+NH4	449.3141236	450.3214	0.000202	3.38	2
Acetyl-L-carnitine	C9H17NO4	M + H	203.11575803	204.12303	−0.03	3.75	2
Sphingofungin A	C21H41N3O6	M + H, M + K, M + Na, M+NH4	431.299537	432.30678	−0.055516	3.38	2
1-alpha dihydroxy diphenyl dinorvitamin D3	C37H48O3	M + Na	540.360346	563.34940	−0.035324	3.68	2
Iohexol	C19H26I3N3O9	M + H	820.8803	821.88748	−0.147865	4.20	2

*MSI level as defined by Sumner et al. (Sumner et al., 2007)

### Improved compound identification giving insights into the metabolomes of patients with COVID-19

**Fig. 6:**
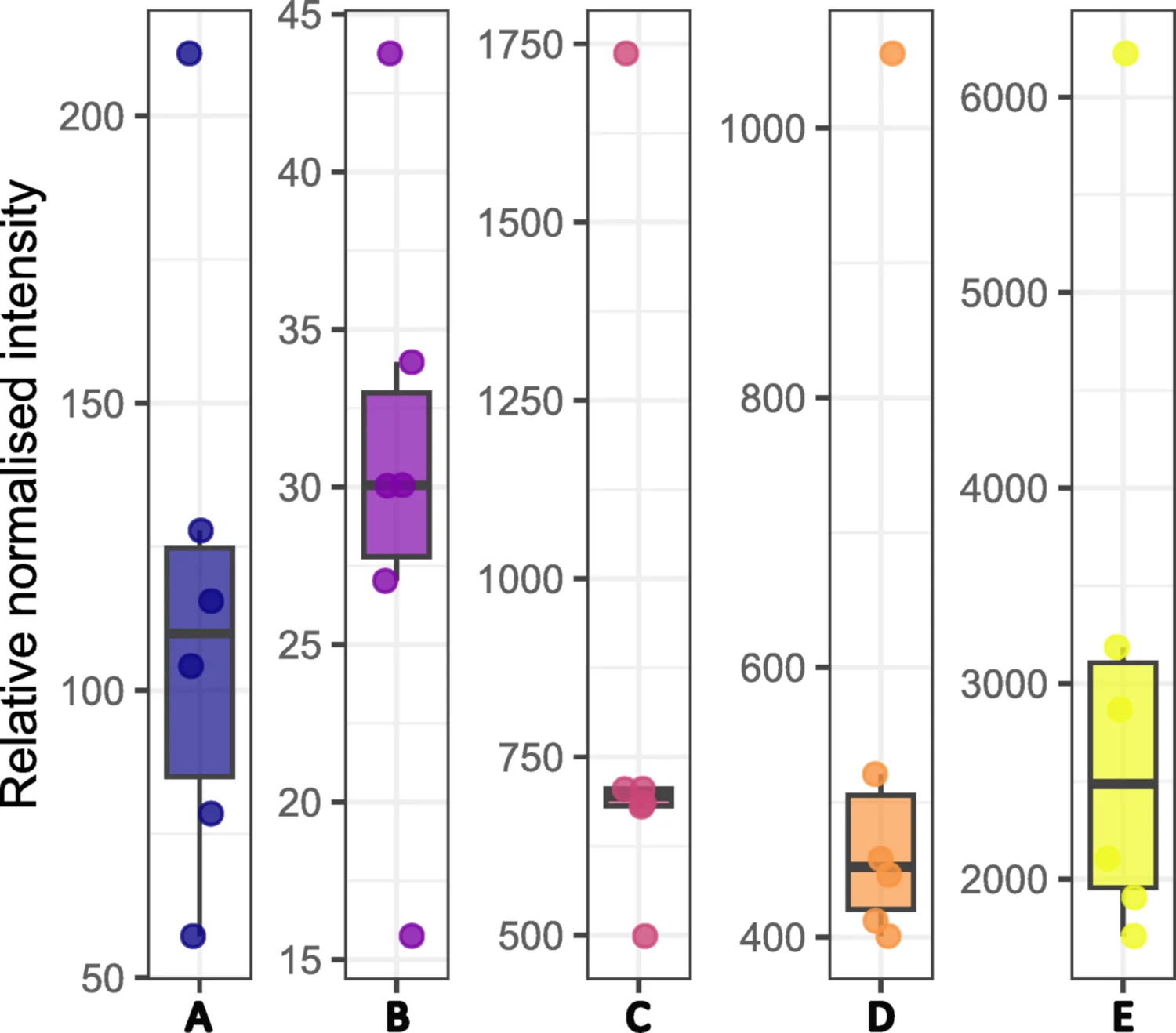
A boxplot describing five of the metabolite features identified which were unique to patient 1. The features in each panel are annotated as follows, with more detail in Table 3;** A** 3.24_579.2535n, annotated as taurochenodeoxycholate sulphate;** B** 3.38_449.3141n, annotated as glycoursodeoxycholic acid;** C** 3.39_431.3036n, annotated as Sphingofungin A;** D** 3.69_563.3512 m/z, annotated as 1-alpha dihydroxy di-phenyl dinorvitamin D3;** E** 4.20_821.8875 m/z, annotated as Iohexol

The improved quality of compound identification using the MRT platform facilitated characterisation of the serum metabolome of seven patients, five with active, severe infections, one with a mild active infection and one who was convalescent form a mild infection. A number of features were annotated as lipids often found in membranes (Supplementary Table S4) and included two odd chain lipids (PE(41:2) and PC(35:1) the origin of which and significance for human health is a topic of research (Venn-Watson et al., 2020). In addition to lipids, Gliclazide, a drug used to treat type 2 diabetes mellitus, was identified in patient 2 whose clinical meta data indicated they had type 2 diabetes mellitus. There were more differences in the serum metabolome of patient 1 compared to the other patients, who revealed that 152 features unique to this individual were identified compared to an average of 37.5 unique features per patient for all other patients. Five of these were annotated and had varying abundancies (Fig. 6; Table 3).

Two of the mass features unique to patient 1could be annotated as bile acid (BA) derivatives – the primary bile acid glycoursodeoxycholic (GUDCA) acid and its metabolite, taurochenodeoxycholate sulphate (TCDC) (Fig. 6A and B). TCDC sulphate is produced first by the conjugation of taurine to the primary bile acidchendeoxycholic acid, followed by sulfation. Conjugation of bile acids with taurine or glycine, together with sulphation, lowers their pKa and improves their solubility in water resulting in more efficient clearance and minimising their reabsorption (Dawson, 2015). In healthy individuals, plasma levels of bile acids are very low, but become elevated in cholestasis where bile acids accumulate in the liver either as a consequence of bile duct blockage or impaired secretion resulting from inflammation. Extremely high plasma bile acids resulting from cholestasis are nephrotoxic and can contribute to renal dysfunction and have previously been evaluated as a predictor of mortality in sepsis (Horvatits et al., 2017; Van Eldere, 2023). Porru and colleagues found that plasma sulphated bile acids were elevated in patients with severe COVID-19 and were a marker of sepsis, but independent of cholestasis and liver damage (Porru, 2024).Clinical metadata for patient 1 indicate they had hypercholesterolemia (Charlat et al., 2025) and kidney failure which are consistent with the annotation of the mass features as bile acids.

Glycoursodeoxycholic acid (GUDCA) (Fig. 6B) is a conjugate of glycine and the secondary bile acid ursodeoxycholic acid (UDCA). UDCA is a hydrophilic bile acid not produced endogenously by humans, but rather by the intestinal microbiome, or alternatively it can be given as a drug intervention to treat cholestatic liver disease (Paumgartner, 2002). It is not clear from the clinical metadata whether UDCA was given as a drug in this case.

A further mass feature was annotated as sphingofungin A (Fig. 6C), a sphingoid metabolite of *Aspergillus fumigatus*, first described by Van Middlesworth and colleagues (Van Middlesworth, 1992). *A. fumigatus* is the most common culprit of COVID-19-associated pulmonary aspergillosis (CAPA), a subtype of invasive pulmonary aspergillosis (IPA) (Salmanton-García, 2021), an opportunistic infection affecting individuals with compromised immunity, particularly where the normal antifungal immunity of the lung is compromised by pulmonary infection and hyperinflammation (Feys, 2024). Fungal biomarkers of IPA and CAPA and have been identified as (1–3)-β-d-glucan (BDG) (commonly described as “Aspergillus antigen”) and galactomannan (GM), which are detectable in serum (Huang, 2024; Rohan, 2024). It is not clear if patient 1 had CAPA, as diagnosis can be challenging (Borman, 2020). However, it has been estimated that 1 in three mechanically ventilated patients had putative IPA (Alanio et al., 2020) and it is a strong possibility that patient 1, who was mechanically ventilated had CAPA. The increasing sensitivity and mass accuracy of model mass spectrometers, such as the MRT platform described here, have the potential to provide much needed better biomarkers of IPA as current test options are limited and lack sensitivity (Huang, 2024; Rohan, 2024).

Another feature was annotated as 1-alpha dihydroxy diphenyl dinorvitamin D3, (Fig. 6D) a synthetic analogue of 1α,25-dihydroxyvitamin D3 (calcitriol) (Maestro, 2019) whilst a fifth was annotated as iohexol (Fig. 6E), a commonly used contrast agent used in imaging procedures such as X-ray and CT scans (Lardieri, 2023; Siegle, 1997). It is generally expected that iohexol is excreted entirely in urine within 24 h of injection, so detection of ioxhexol in serum implies very recent contrast imaging has been undertaken (Olsson, 1983). Iohexol was abundant in serum from patient 1 who had a CT scan several days prior to donating the serum sample. Clinical meta data indicated that patient 1 had recently had a CT scan and had kidney failure, which is documented to delay iohexol clearance (Delanaye et al., 2016), could explain the finding of a feature which could be annotated as iohexol.

## Conclusions

The accurate identification of features of interest is attracting significant interest with the metabolomics and, lipidomics communities, recent publication by Theodoridis et-al (Theodoridis, 2023) illustrated the challenges faced in ensuring fact-based metabolite identification. The MRT MS platform described here, with its high MS resolution, > 200,000 FWHM, and high-speed (10–20 Hz) data acquisition, facilitated the collection of high resolution “fine isotope” MS data at acquisition speeds which allowed the accurate identification of lipids and metabolites in complex “real world” patient samples. It showed the ability to acquire the fine isotopic detail. This allowed the resolution and identification of overlapping lipid species which would otherwise have gone undetected with a conventional ToF or electrostatic trap type instrument. The ability to provide high mass accuracy for compound identifications, significantly limits the search space and therefore reduces the number of false positives reported.

The platform enabled identification of compounds present in the circulation of a critically ill patient with COVID-19 which gave insights into their condition which were consistent with the patient’s clinical history. Whilst this study was undertaken in a only a small subset of the larger ManARTS COVID-19 cohort its offers a proof-of-principle demonstration of the application of the MRT platform to undertake metabolite profiling in “real-world” samples, which are complicated by numerous uncontrolled variables. Being able to undertake such detailed analysis at scale and in a rapid manner is essential to unravelling the dysregulation of metabolism in disease to identify both diagnostic and prognostic biomarkers as well as therapeutic targets. Whilst this small study does not fully address the challenges associated with larger metabolomics studies, the high-quality MS1 and MS2 spectra achieved in this study shows how technology, such as the MRT platform, can significantly improve the chances of accurate feature identification whilst allowing the high throughput analysis required for well-powered, large cohorts from clinical and epidemiological studies.

## Supplementary Information

Below is the link to the electronic supplementary material.

Supplementary Material 1

Supplementary Material 2

Supplementary Material 3

## Data Availability

The metabolomic data and metadata are available on the MetaboLights public database [ebi.ac.uk/metabolights], study identifier REQ20260603220293.
