# Supplementary material for: Multi-reflecting time-of-flight mass spectrometry enables high-accuracy metabolite identification in a demonstration study using serum samples from COVID-19 patients
Source: Metabolomics. 2026 Oct 3;22(5):168. doi: 10.1007/s11306-026-02539-w (PMC13633998; doi:10.1007/s11306-026-02539-w)
Supplement: Supplementary file 1 [file 11306_2026_2539_MOESM1_ESM.docx]

**Multi-Reflecting Time-of-Flight Mass Spectrometry Enables High-Accuracy Metabolite Identification - a Demonstration Study using Serum Samples from COVID-19 Patients**

Adam King ^1^, Eleanor Matthews ^2^, Garth Maker ^3^, Robert Trengrove ^4^, Shaufa Shareef ^2^, Stephen Fowler^2,5^, Timothy Felton^2,5^, Angela Simpson^2,5^, Martin Palmer ^1^, Lee A Gethings ^1,2,6^, Robert S Plumb ^7^, and E. N. Clare Mills ^1,6*^

1 Waters Corporation, Wilmslow, Cheshire, SK9 4AX, United Kingdom; adam.king@waters.com; mar-tin_palmer@waters.com, lee_gethings@waters.com

2 Division of Infection, Immunity and Respiratory Medicine, University of Manchester, Manchester, United Kingdom; eleanor.matthews-3@postgrad.manchester.ac.uk; aishathshaufa.shareef@manchester.ac.uk; lee.gethings@manchester.ac.uk; clare.mills@manchester.ac.uk ,angela.simpson@manchester.ac.uk;

3 Medical and Molecular Sciences, School of Veterinary and Life Sciences, Murdoch University, South Street, Murdoch, WA 6150, Australia; [g.maker@murdoch.edu.au]

4 Curtin Health Innovation Research Institute, Curtin University, Kent St, Bentley, WA 6102, Australia; [rob-ert.trengove@curtin.edu.au]

5 Wythenshawe Hospital, Manchester University NHS Foundation Trust, Manchester M23 9LT, Greater Manchester, UK

6 School of Biosciences,, University of Surrey, Guildford, United Kingdom; clare.mills@surrey.ac.uk

7 Waters Corporation, Milford, MA, 01757, USA; rob_plumb@waters.com

*Correspondence: [clare.mills@surrey.ac.uk](mailto:clare.mills@surrey.ac.uk)

**Supplementary Information**

**Contents**

[Supplementary Table S1: Root mean square accurate mass measurements representing therapeutic xenobiotics and metabolites acquired over a 24h period.	3](#_Toc240312516)

[Supplementary Table S2. Root mean square accurate mass measurements representing therapeutic xenobiotics and metabolites acquired over a 24 h period.	4](#_Toc240312517)

[Supplementary Figure S1: Patient disease journeys	3](#_Toc240312518)

[Supplementary Figure S2. Schematic of the SELECT SERIES MRT flight tube.	8](#_Toc240312519)

[Supplementary Table S3: Batch design table	9](#_Toc240312520)

[Supplementary Table S4: Data cleaning overview	10](#_Toc240312521)

[Supplementary Methods S1:	11](#_Toc240312522)

[Supplementary Figure S3: Unsupervised Principal Components Analysis plot	12](#_Toc240312523)

[Supplementary Figure S4: Coefficients of variance distributions in the pooled QC sample.	13](#_Toc240312524)

[Supplementary Figure S5. Fine isotopic pattern of lipid features with potassium adducts.	14](#_Toc240312525)

[Supplementary Table S4: Compound annotation in serum metabolomics of patients 2, 4-8.	15](#_Toc240312526)

[Supplementary Table S5: Matched fragments for Sphingofungin A annotation	16](#_Toc240312527)

[References	16](#_Toc240312528)

# **Supplementary Table S1: Root mean square accurate mass measurements representing therapeutic xenobiotics and metabolites acquired over a 24h period.**

Data were acquired is positive ionisation mode. RMS - Root mean square; retention time is in minutes. Urine samples were taken from human subjects 0, 2, 4, and 6 hours after administered of medication (Carbamazepine [2 x 200 mg], acetaminophen [2 x 500 mg] or Naproxen [1 x 500 mg]) and diluted 1:10 (v:v) prior to analysis on a SELECT SERIES MRT equipped with an ACQUITY Ultra Performance LC™ and a ACQUITY UPLC HSS T3 C_18_ (100 mm X 2.1 mm, 1.8 µm) column. A metabolite identification workflow using LC-MS (system resolution >200,000 FWHM) has been implemented.^4^. Data are from McCullagh M., Palmer M., Eatough, D., Marsden-Edwards E., Langridge, J. I., Cooper-Shepherd D. Waters Application Note [Exploring the Impact of Part Per Billion Mass Accuracy for Metabolite Identification Using Multi Reflecting Time-of-Flight MS With UPLC™ Part A](https://www.waters.com/content/dam/waters/en/app-notes/2023/720007896/720007896-en.pdf) (<https://www.waters.com/nextgen/us/en/library/application-notes/2023/exploring-the-impact-of-part-per-billion-mass-accuracy-for-metabolite-identification-using-multi-reflecting-time-of-flight-ms-with-uplc-part-a.html>).

# **Supplementary Table S2. Root mean square accurate mass measurements representing therapeutic xenobiotics and metabolites acquired over a 24 h period.**

Data were collected as described for Table S1 except they were acquired in negative ionisation mode.

# **Supplementary Figure S1: Patient disease journeys**

**Patient 1**

**Patient 2**

**Patient	3**

**Patient	4**

**Patient 5**

**Patient 6**

**Patient 7**

**Patient 8**

**Supplementary Figure S2. Schematic of the SELECT SERIES MRT flight tube.**

# **Supplementary Table S3: Batch design table**

One biological sample (an aliquot of serum) was obtained for each patient. This was extracted in duplicate to produce two independent extraction replicates. Each of these extraction replicates was injected in triplicate, to produce a total of six technical replicates.

| **Injection number** | **Sample** |  | **Injection number (cont.)** | **Sample** |
| --- | --- | --- | --- | --- |
| 1 | Blank |  | 31 | Patient 5, extraction B |
| 2 | Pooled QC |  | 32 | Patient 1, extraction B |
| 3 | Pooled QC |  | 33 | Patient 3, extraction A |
| 4 | Pooled QC |  | 34 | Patient 7, extraction B |
| 5 | Pooled QC |  | 35 | Patient 8, extraction A |
| 6 | Pooled QC |  | 36 | Patient 2, extraction B |
| 7 | Pooled QC |  | 37 | Patient 2, extraction A |
| 8 | Blank |  | 38 | Patient 8, extraction A |
| 9 | Patient 4, extraction A |  | 39 | Pooled QC |
| 10 | Patient 6, extraction A |  | 40 | Patient 2, extraction A |
| 11 | Patient 4, extraction A |  | 41 | Patient 7, extraction A |
| 12 | Patient 4, extraction A |  | 42 | Patient 2, extraction A |
| 13 | Patient 6, extraction B |  | 43 | Patient 4, extraction B |
| 14 | Patient 6, extraction A |  | 44 | Patient 2, extraction B |
| 15 | Patient 8, extraction B |  | 45 | Patient 4, extraction B |
| 16 | Patient 1, extraction B |  | 46 | Patient 4, extraction B |
| 17 | Pooled QC |  | 47 | Patient 1, extraction B |
| 18 | Patient 3, extraction A |  | 48 | Patient 6, extraction B |
| 19 | Patient 5, extraction A |  | 49 | Patient 5, extraction A |
| 20 | Patient 7, extraction A |  | 50 | Pooled QC |
| 21 | Patient 3, extraction A |  | 51 | Patient 2, extraction A |
| 22 | Patient 7, extraction A |  | 52 | Patient 6, extraction B |
| 23 | Patient 8, extraction B |  | 53 | Patient 7, extraction B |
| 24 | Patient 6, extraction A |  | 54 | Patient 8, extraction B |
| 25 | Patient 5, extraction A |  | 55 | Patient 7, extraction B |
| 26 | Patient 1, extraction A |  | 56 | Patient 8, extraction A |
| 27 | Patient 1, extraction A |  | 57 | Patient 5, extraction B |
| 28 | Patient 2, extraction B |  | 58 | Pooled QC |
| 29 | Pooled QC |  | 59 | Blank |
| 30 | Patient 5, extraction B |  |  |  |

**Supplementary Table S4: Data cleaning overview**

| **Stage** | **Activity** | | | **Total features eliminated** | **Total features remaining** |
| --- | --- | --- | --- | --- | --- |
| **Comparisons between patients** | | | | | |
| 1 | Progenesis QI processing | | | 0 (0 %) | 7,458 (100 %) |
| 2 | Pooled QC sample analysis | | No. of features in pooled QC: 3,554 | 3,904 (52.3 %) | 3,554 (47.7 %) |
|  |  |  | No. of features with a Min %CV < 30: 758 | 6,700 (89.8 %) | 758 (10.16 %) |
| 3 | Replication analysis | | No. of features present in at least 2 out of 3 injections of either extraction replicate | 6,700 (89.8 %) | 758 (10.16 %) |
|  |  |  | No. of features present in 2 out of 2 extraction replicates of each sample | 6,722 (90.1 %) | 736 (9.9 %) |
| **Analysis of Patient 1 only** | | | | | |
| 1 | Progenesis QI processing | | | 0 (0 %) | 7,458 (100 %) |
| 2 | Min %CV analysis | No. of features with a Min %CV < 30 across all technical replicates of both of Patient 1’s extraction replicates | | 6,330 (84.86 %) | 1,128 (15.12 %) |
| 3 | Replication analysis | No. of features appearing in at least 2 out of 3 injections of either of Patient 1’s extraction replicates | | 6,331 (84.88 %) | 1,127 (15.11 %) |
|  |  | No. of features appearing in both of Patient 1’s extraction replicates | | 6,590 (88.72 %) | 868 (11.64 %) |

**Supplementary Methods S1:**

For the heatmap in Figure 2, technical replicates were first collapsed by taking the median normalised abundance measurement for each detected feature to represent their extraction replicate. The same strategy was then used to collapse the two extraction replicates to provide a single value per feature per patient, thereby avoiding psuedoreplication for this analysis. The resulting dataset was used to evaluate the features by their variability between mildly ill patients (n = 2) and severely ill patients (n = 5). This was done conducting a Wilcoxon rank-sum test for each feature. A Benjamini-Hochberg correction was then performed to correct for multiple testing, providing a false discovery rate adjusted p-value for each feature. The 100 features with the lowest p-values (ranging from 0.030 – 0.081) were selected. The FDR-adjusted p-values (Benjamini-Hochberg q-values) for these were all 0.38. These features are displayed in a heatmap, with replicates un-collapsed for transparency. No strict cut-off values were applied as the analysis we have conducted is purely exploratory, and the statistical analyses applied were for the purpose of prioritising features for visualisation.

The heatmap was produced using Metaboanalyst 6.0 using the following settings; “autoscale features” was selected, meaning that normalised abundance values for each feature were converted into Z-scores so that every feature is weighted equally. Dendrogram grouping of features was done on the basis of Euclidean Distance. Clustering of samples was not performed. Feature clustering was performed using Ward’s method.

For the boxplots in Figure 6, each point represents a technical replicate. These replicates were not collapsed and are treated as independent observations.

# **Supplementary Figure S3: Unsupervised Principal Components Analysis plot**

Patient serum samples are numbered 8 with the replicates for each patient denoted by a different colour; QC -pooled quality control sample comprising serum from all patients. Each point represents an injection (technical replicate). All 6 technical replicates for each patient sample (3 per extraction replicate) are plotted. Data are presented for samples analysed for all patients except patient 3 which was excluded following the initial data preprocessing (see Supplementary Table S4).

**Supplementary Figure S4: Coefficients of variance distributions in the pooled QC sample.**

Histograms showing the distribution of coefficient of variance (CV) as a percentage, in the dataset before (A) and after (B) cleaning. Bin sizes are 1 and 20 respectively. (See also Supplementary Datasheet S1).

A

B

**Supplementary Figure S5. Fine isotopic pattern of lipid features with potassium adducts**.

(A) isotopic distributions the feature annotated as PC (34:2) [M+K]+ adduct; (B) the full isotopic distribution for lipid m/z = 798.54163 over an expanded region highlighting the 39K, 41K and 13C2 isotopes of the PC (34:2) [M+K]+ (indicated in green) and lipid m/z = 796.52606 alongside the monoisotopic peak for m/z = 798.54163; (C) shows the raw isotopic distribution data (red) overlaid with modelled theoretical fine isotopic pattern for lipid m/z = 796.52606 (green) and modelled monoisotopic peak for lipid m/z = 798.54163 (blue).

**(**

**Supplementary Table S4: Compound annotation in serum metabolomics of patients 2, 4-8**.

Analysis was undertaken in positive ionisation mode.* MSI level as defined by (Sumner et al., 2007).

| **Annotation** | **Elemental formula** | **Adduct** | **Neutral Mass (Da)** | **m/z** | **Mass error (ppm)** | **Retention time (min)** | **MSI level*** | **Patients sample** | **Biological significance** |
| --- | --- | --- | --- | --- | --- | --- | --- | --- | --- |
| PE(28:4) | C43H78NO8P | M+H, M+K | 627.3900 | 767.5465n | 0.01 | 1.70 | 2 | Patients 4, 5, 6, 8 | Membranes lipid |
| PE(41:2) | C46H88NO8P | M+H | 813.6248 | 814.6321 | 0.04 | 1.49 | 2 | Patients 2,4,5,6,8 | Odd chain lipid |
| PE-NMe(34:2) | C40H76NO8P | M+H, M+K | 729.5309 | 729.5309n | 0.03 | 1.57 | 2 | Patients 2,4,5,6,8 | Enriched in mitochondrial membranes |
| PC(35:1) | C43H84NO8P | M+H | 773.5935 | 774.6007 | -0.01 | 1.51 | 2 | Patients 4, 5, 6, 8 | Odd chain lipid |
| PC(P-34:1) | C42H82NO7P | M+H | 743.5829 | 744.5902 | 0.03 | 1.51 | 2 | Patient 6 | Plasmalogen phospholipid involved in combating oxidative stress. |
| Cer(42:2) | C42H81NO3 | M+H, M+K | 647.6216 | 647.6217n | 0.07 | 0.36 | 2 | Patient 6 | Sphingolipid with role in orchestrating inflammatory responses |
| Tricosanedioic acid | C23H44O4 | M+NH4 | 384.32396 | 402.3578 | 0.07 | 0.86 | 2 | Patient 8 | Long-chain dicarboxylic fatty acid generated through ω-oxidation of fatty acids. |
| Gliclazide | C15H21N3O3S | M+H, M+K,  M+Na | 323.13036 | 323.1304n | 0.23 | 0.39 | 2 | Patient 2 |  |

**Supplementary Table S5: Matched fragments for Sphingofungin A annotation**

The Progenesis MetaScope score for the feature is 42.7/80 with a fragmentation score for this feature of 35. The score is based on mass similarity, isotope distribution similarity, retention time similarity, and fragmentation score. No alternative candidate structures were identified for this feature.

| **Fragment m/z** | **Peak m/z** | **Fragment formula** |
| --- | --- | --- |
| 177.1041 | 177.1069 | C10H19+K |
| 414.2963 | 414.3003 | C21H40N3O5-e |
| 454.2887 | 454.2927 | C21H41N3O6+Na |
| 470.2627 | 470.2666 | C21H41N3O6+K |

# **References**

Sumner, L. W., Amberg, A., Barrett, D., Beale, M. H., Beger, R., Daykin, C. A., Fan, T. W., Fiehn, O., Goodacre, R., Griffin, J. L., Hankemeier, T., Hardy, N., Harnly, J., Higashi, R., Kopka, J., Lane, A. N., Lindon, J. C., Marriott, P., Nicholls, A. W.,…Viant, M. R. (2007). Proposed minimum reporting standards for chemical analysis Chemical Analysis Working Group (CAWG) Metabolomics Standards Initiative (MSI). *Metabolomics*, *3*(3), 211–221. <https://doi.org/10.1007/s11306-007-0082-2>
